# Reply: Do we need biopsies of metastases for colorectal cancer patients?

**DOI:** 10.1038/sj.bjc.6605170

**Published:** 2009-07-07

**Authors:** M Frattini, F Molinari, P Saletti, L Mazzucchelli

**Affiliations:** 1 Institute of Pathology, Via in Selva 24, 6600 Locarno, Switzerland; 2 Oncology Institute of Southern Switzerland, Ospedale San Giovanni, Bellinzona, Switzerland

**Sir,**

We thank Dr Floriani and co-workers for their interest in our work and for their stimulating comments. It is important to highlight that so far in metastatic colorectal cancer patients, treatment with cetuximab or panitumumab is accomplished to block or to destroy metastatic cells, whereas the evaluation of molecular markers able to predict the efficacy of these therapies has been performed mostly on primary tumours. Historically, the choice of researchers to use primary tumours for molecular studies was made because biopsies of the metastatic lesions were not available in the majority of patients. As a consequence, it is reasonable to hypothesise that primary tumours and related distant metastatic lesions may show different molecular fingerprints that may affect the prediction of response to targeted therapies.

We agree with Floriani and co-workers that K-Ras is the only universally accepted determinant of resistance to EGFR-targeted drugs. However, recent pre-clinical (Di Nicolantonio *et al*, 2008; Jhawer *et al*, 2008) and clinical (Frattini *et al*, 2007; Di Nicolantonio *et al*, 2008; Sartore-Bianchi *et al*, 2009) data show that other members of the EGFR downstream pathways (e.g., BRAF, PTEN and PIK3CA) may have a predictive role in this context. We found a rationale for our studies as no previous works reported a simultaneous comparison of these markers in the same cohort of patients. To evaluate the discrepancies between primary tumour and paired distant metastatic lesions, we applied Cohen's kappa test, one of the most used and solid tests for this type of work. Our results are in line with the few data available in the literature on this topic and, although not statistically significant, the results suggest that differences between primary tumour and metastases may be clinically relevant for a given patient. We are therefore pleased that Floriani and co-workers after reading our work came to the same conclusions, namely that prospective studies on this important topic are extremely urgent.
